# ARTHROSCOPIC TREATMENT OF OSTEOCHONDRAL LESIONS OF THE TALUS

**DOI:** 10.1590/1413-785220162401137414

**Published:** 2016

**Authors:** Mariana Korbage de Araujo, Mario Sergio Paulillo de Cillo, Cinthia Kelly Bittar, José Luis Amin Zabeu, Caroliny Nociti Moreira Cezar

**Affiliations:** 1. Pontifícia Universidade Católica de Campinas, Hospital e Maternidade Celso Pierro, Department of Orthopedics and Traumatology, Campinas, SP, Brazil.

**Keywords:** Ankle, Arthroscopy, Talus, Wounds and injuries

## Abstract

**Objective:**

To assess pain and function of the ankle in patients with injuries up to 1.5 cm diameter by the American Orthopaedic Foot and Ankle Society (AOFAS) score after arthroscopic treatment.

**Methods:**

The AOFAS scale was applied before and after arthroscopy, as well as the degree of subjective satisfaction of ambulatory patients. Patients with type I osteochondral injuries, acute trauma, using plaster, presenting lesions in other joints of the lower limbs and cognitive impairment that would prevent the application of the satisfaction questionnaire were excluded from the study. Statistical analysis was performed using unpaired t test with Welch correction, Mann Whitney test, and ANOVA, with Kruskal Wallis test and Dun test, considering p value lower than 0.05.

**Results:**

There was an increased AOFAS scores after arthroscopic treatment in 52 (94.5%) patients. The mean values of AOFAS score in 55 patients was 77.32 ± 6.67 points preoperative and 93.10± 8.24 points postoperative, with a mean variation of 15.8 points, p<0.001. Patients with stage II, III and IV injuries showed an increased AOFAS scores after arthroscopic treatment, p<0.001. No difference was found between medial and lateral injuries, p >0.05.

**Conclusion:**

Patients with stage II, III or IV osteochondral injuries of the talus of up to 1.5 cm diameter, whether medial or lateral, showed a significant improvement after arthroscopic treatment.*** Level of Evidence III, Retrospective Study.***

## INTRODUCTION

Osteochondral injuries of the talus account for 1% of body fractures which often represent late sequelae of trauma to the ankle and evolve with loss of movement and local arthrosis, besides a significant impact on the patient's quality of life.^1^ ^-^ ^4^ The most used classification of osteochondral injuries proposed by Berndt and Harty^5^ in 1959 is radiological. However, other classifications using computed tomography (CT) and magnetic resonance imaging (MRI) have also been proposed.^6^ ^,^ ^7^ The treatment of osteochondral injuries of the talus is based on size, location and extent of the injury. Injuries stage I can be treated conservatively.^4^ ^,^ ^8^ Stage II, III and IV injuries may be surgically treated by arthroscopy, arthrotomy, mosaicplasty or chondrocyte culture implant.^4^

The arthroscopic technique allows precise access to the site of injury and less tissue damage, allowing faster and functional recovery with fewer complications inherent to arthrotomy such as infection, neurovascular injury, osteoarthritis and improper posterior visualization.^4^ ^,^ ^9^ ^-^ ^11^ Injuries up to 1.5 cm appear to have better results by arthroscopy than larger injuries.^9^ ^,^ ^12^

The objective of this study was to assess pain and function of the ankle after arthroscopic treatment of patients with osteochondral injuries of the talus up to 1.5 cm diameter in ambulatory followup.

## MATERIALS AND METHODS

This study was approved by the Medical Ethics Committee of *Hospital Municipal Mário Gatti*, Campinas, SP, Brazil, under CAAE number 30256814.0.0000.54530. A Free and Informed Consent form was signed by all participants.

The study included skeletally mature patients undergoing arthroscopy of the talus from November 1997 to August 2010, presenting osteochondral injuries of the talus up to 1.5 cm diameter. Injuries were classified radiologically according to Berndt and Harty^5^ and their location as anterolateral, anteromedial, posteromedial and posterolateral. Computed tomography (CT) and magnetic resonance imaging (MRI) were also used to assist the classification and evaluation of the integrity of the lateral ankle ligaments.

Arthroscopic treatment was performed by excision, debridement and perforation^4^ ^,^ ^7^ ^,^ ^9^ ^,^ ^13^ ^,^ ^14^ and the patient was instructed not to perform physical efforts in the first 30 days and return to sports practice 12 weeks after the surgery.

The scale of the American Orthopaedic Foot and Ankle Society (AOFAS)^15^ was applied preoperatively and at outpatient follow-up. The location and the stage of the injuries were considered. Subjective satisfaction questionnaire were also applied one year after the procedure, considering the following responses: Very satisfied; satisfied; regular or dissatisfied. The researchers who applied the AOFAS scale and satisfaction questionnaire did not participate at the arthroscopy procedures.

Patients with osteochondral injuries type I, patients with acute trauma, using plaster, injuries in other joints of the lower limbs and cognitive alterations that did not allow application of the satisfaction questionnaire were excluded from the study. In cases with complications and those requiring more than one arthroscopy, only the first evaluation procedure was considered.

Statistical analysis was performed using the *t* test and ANOVA analysis of variance and unpaired values with Kruskal-Wallis and Dun test, considering as significant *p*<0.05. 

## RESULTS 

Sixty five patients were analyzed and 10 were excluded. Among the 55 patients included, 34 (61.8%) were men and 21 (38.2%) were women. The age of patients at the time of surgery ranged from 17 to 57 years old, mean 36.89 ± 9.15 years old.

We identified 30 (54.5%) injuries on the right side, 24 (43.6%) on the left side and one (1.9%) bilateral injury.

Among the 34 medial injuries, 11 (32.3%) were ranked as stage II, 20 (58.8%) as stage III, three (8.8%) as stage IV. Among the 21 lateral injuries, 10 (47.6%) were stage II, 10 (47.6%) were stage III and one (4.8%) stage IV. Central injuries were not found.

The postoperative follow-up of these patients ranged from 10 to 48 months, 20.23 ± 8.6 months, on average.

Table 1 shows the distribution of mean AOFAS scores at pre and postoperative periods according to the stage of the injury.

**Table 1:** Distribution of mean AOFAS scores according to the stage of the injury.

Stage of the injury (n)	Preoperative AOFAS mean ± sd	Postoperative AOFAS mean ± sd	Difference	*p*
II (22 )	79.09 ± 7.71	95.19 ± 6.17	16.1	<0.001
III (30 )	76.66 ± 6.0	91.16 ± 9.23	14.5	<0.001
IV (4)	73.0 ± 2.0	96.75 ± 6.5	23.7	=0.003
Total (55)	77.32 ± 6.67	93.10 ± 8.24	15.8	<0.001

No difference was observed between medial and lateral injuries, p> 0.05, nor between anterior and posterior injuries, p> 0.05. Table 2 shows the distribution of average AOFAS scores in the pre and postoperative periods according to the location of the injury.

**Table 2:** Distribution of mean AOFAS scores according to the location of the injury.

Stage of the injury (n)	Preoperative AOFAS mean ± sd	Postoperative AOFAS mean ± sd	Difference	*p*
Posteromedial (28)	77.62 ± 6.86	93.25 ± 9.06	15.5	<0.001
Anterolateral (17)	76.76 ± 7.62	93.80 ± 6.80	17.1	<0.001
Anteromedial (6)	78.0 ± 5.44	91.51 ± 6.77	13.5	0.002
Posterolateral (4)	74.75 ± 0.95	89.75 ± 11.95	15.0	0.04
Medial total (34)	77.91 ± 6.57	93.14 ± 8.58	15.5	<0.001
Lateral total (21)	76.38 ± 6.87	93.04 ± 7.87	16.6	<0.001
Anterior total (23)	77.08 ± 7.01	93.03 ± 6.77	17.4	<0.001
Posterior total (32)	77.50 ± 6.51	93.03 ± 9.26	14.1	<0.001

Subjective assessment of postoperative patients revealed 51 (92.7%) of satisfactory cases. Table 3 shows the distribution of mean AOFAS scores according to the subjective degree of patient's satisfaction.

**Table 3:** Distribution of mean AOFAS scores according to patient's degree of satisfaction.

Degree of satisfaction (n)	Preoperative AOFAS mean ± sd	Postoperative AOFAS mean ± sd	*p*
Very satisfied (19)	81.25 ± 5.42	99.84 ± 0.68	<0.001
Satisfied (22)	71.41 ± 5.78	91.25 ± 6.19	<0.001
Not satisfied (4)	72.25 ± 9.81	76.00 ± 10.48	0.3119

In this study, 42 (76.4%) patients were older than 30 years at the time of surgery, with an average of pre- or postoperative values ​​by AOFAS scale not different from patients younger than 30 years, p> 0.05. Similarly, men showed no significant difference from women in the AOFAS assessment, *p*>0.05. 

## DISCUSSION

This is a cohort retrospective non controlled study which evaluated pain and ankle function before and after treatment of osteochondral injuries of the talus up to 1.5 cm diameter by arthroscopic technique.

Osteochondral injuries of the talus were more frequent in men, located mostly on the right side, at the medial portion with predominance of posteromedial injuries, which was also found in other studies.^9^ ^,^ ^10^ ^,^ ^16^ ^,^ ^17^ The low prevalence of stage IV injuries is also in accordance with other studies.^9^

This study demonstrated a significant improvement of pain and ankle function after treatment of osteochondral injuries of the talus by arthroscopy, assessed by the AOFAS scale. Tol et al.^18^ found 88% good and excellent results in patients with stage III injuries or more advanced injuries who underwent arthroscopy under the same conditions as the present study. Chuckpaiwong et al.^12^ found 100% success in injuries smaller than 1.5 cm diameter, although another study^9^ associated better results to injuries smaller than 1 cm. Guo et al.^9^ also found significant improvement, with an average score of 90.16 ± 9.96 postoperatively as compared to 70.81 ± 6.96 preoperatively by the AOFAS scale.

The improvement of pain and ankle function was significant in both the medial injuries as the lateral ones. Twenty three (67.6%) injuries of the 34 medial injuries were stage III or IV. Eleven injuries (52.3%) of the 21 lateral injuries were stage III or IV without significant difference, p> 0.05. Schimmer et al.^17^ found better results with medial injuries, but only 26.3% of them were stage III or IV. Guo et al.^9^ found no differences between medial or lateral injuries, and all lateral injuries and 86% medial injuries were stage III or IV.

The subjective degree of patient satisfaction was not significant for stage IV injuries. Guo et al.,^9^ using a visual scale, found good correlation between AOFAS score and patient's visual scale. Ogilvie-Haris and Sarrosa^14^ observed improvement of pain, swelling, stiffness, limping and physical activity after arthroscopy.

## CONCLUSION

Treatment of osteochondral injuries of the talus up to 1.5 cm diameter by arthroscopy and injury resection with drilling and debridement improves pain and function of the ankle, regardless of the stage of the injury and its medial or lateral location.
